# Cigarette smoke stimulates clonal expansion of Jak2^V617F^ and Tet2^-/-^ cells

**DOI:** 10.3389/fonc.2023.1210528

**Published:** 2023-07-21

**Authors:** Gajalakshmi Ramanathan, Jane H. Chen, Nitya Mehrotra, Tiffany Trieu, Aaron Huang, Eduard Mas, Jessica E. Monterrosa Mena, Bishop Bliss, David A. Herman, Michael T. Kleinman, Angela G. Fleischman

**Affiliations:** ^1^Department of Medicine, Division of Hematology/Oncology, University of California, Irvine, Irvine, CA, United States; ^2^Department of Medicine, Division of Occupational and Environmental Medicine, University of California, Irvine, Irvine, CA, United States; ^3^Chao Family Comprehensive Cancer Center, University of California, Irvine, Irvine, CA, United States

**Keywords:** inflammation, myeloproliferative neoplasm, reactive oxygen species, cigarette smoke, clonal hematopoiesis

## Abstract

**Introduction:**

Somatic mutations in myeloid growth factor pathway genes, such as JAK2, and genes involved in epigenetic regulation, such as TET2, in hematopoietic stem cells (HSCs) leads to clonal hematopoiesis of indeterminate potential (CHIP) which presents a risk factor for hematologic malignancy and cardiovascular disease. Smoking behavior has been repeatedly associated with the occurrence of CHIP but whether smoking is an environmental inflammatory stressor in promoting clonal expansion has not been investigated.

**Methods:**

We performed in vivo smoke exposures in both wildtype (WT) mice and transplanted mice carrying Jak2^V617F^ mutant and Tet2 knockout (Tet-/-) cells to determine the impact of cigarette smoke (CS) in the HSC compartment as well as favoring mutant cell expansion.

**Results:**

WT mice exposed to smoke displayed increased oxidative stress in long-term HSCs and suppression of the hematopoietic stem and progenitor compartment but smoke exposure did not translate to impaired hematopoietic reconstitution in primary bone marrow transplants. Gene expression analysis of hematopoietic cells in the bone marrow identified an imbalance between Th17 and Treg immune cells suggesting a local inflammatory environment. We also observed enhanced survival of Jak2V617F cells exposed to CS in vivo and cigarette smoke extract (CSE) in vitro. WT bone marrow hematopoietic cells from WT/Jak2V617F chimeric mice exposed to CS demonstrated an increase in neutrophil abundance and distinct overexpression of bone marrow stromal antigen 2 (Bst2) and retinoic acid early transcript 1 (Raet1) targets. Bst2 and Raet1 are indicative of increased interferon signaling and cellular stress including oxidative stress and DNA damage, respectively. In chimeric mice containing both WT and Tet2-/- cells, we observed an increased percentage of circulating mutant cells in peripheral blood post-cigarette smoke exposure when compared to pre-exposure levels while this difference was absent in air-exposed controls.

**Conclusion:**

Altogether, these findings demonstrate that CS results in an inflamed bone marrow environment that provides a selection pressure for existing CHIP mutations such as Jak2V617F and Tet2 loss-of-function.

## Introduction

Clonal hematopoiesis (CH) describes the dominance of a specific hematopoietic stem cell clone in the peripheral blood. Clonal hematopoiesis of indeterminate potential (CHIP), is an age-related condition where hematopoietic stem cells (HSCs) that have acquired somatic mutations give rise to mature mutant cells in the peripheral blood with a variant allele frequency of at least 2% (1–3). CHIP is very common in the elderly with at least 10% of the population over 65 years being affected (1). CHIP not only presents an increased risk for developing hematologic malignancies but is also associated with a range of co-morbidities that involve inflammation such as cardiovascular disease (4, 5) and gout (6). Somatic mutations in recurrent genes confer a selective growth advantage or increased fitness in mutant HSC clones. DNMT3A, TET2, ASXL1 and JAK2 are among the commonly mutated genes associated with CHIP (1–3).

Although CHIP is very common after the fifth decade of life the emergence of CH is variable and may take several years before a pathogenic mutant can overtake its normal counterparts in the stem cell pool (7). Clonal dominance can be accelerated by cell extrinsic factors including inflammation (8), genotoxicity (9), and expediated HSC proliferation (10). Thus, specific environmental stressors may provide ideal conditions for outgrowth of specific mutant clones that are superiorly adapted for the stressor. Age is the strongest determinant for CHIP indicating that inflammatory stress or inflammaging associated with the natural process of aging contributes to CH (11). Environmental influences such as smoking (12) and cytotoxic chemotherapy (13) have also been repeatedly associated with clonal hematopoiesis.

Myeloproliferative neoplasms (MPNs) are characterized by clonal outgrowth of mutant hematopoietic stem cells contributing to the over-production of circulating mature cells of the myeloid lineage (14). MPNs can be described as a human inflammation model of cancer development where chronic inflammation is a hallmark feature of myeloproliferative neoplasms (MPNs) and is associated with disease initiation and progression (15). The most common somatic mutation in MPN patients occurs in Janus Kinase 2 (JAK2) known as the JAK2^V617F^ mutation (16–19). We have shown that an *in vitro* inflammatory environment facilitates the selective expansion of the JAK2*^V617F^* neoplastic clone in human subjects (20). More recently, IL-1b exposure stimulated clonal expansion of Jak2^V617F^ mutant cells and accelerated progression to bone marrow fibrosis (21). Additional extrinsic mechanisms mediating JAK2^V617F^ clonal expansion are lacking. Epidemiological studies such as the UK Million Women Study and the Iowa Women’s Health Study have reported a correlation between smoking and the development MPNs (22, 23). Sørensen and Hasselbalch, also described a positive association between smoking behavior and the risk for MPN (24). It is possible, that the systemic inflammation associated with smoking can support clonal expansion of the JAK2^V617F^ malignant clone.

Biological studies have demonstrated that *Tet2*-deficient hematopoietic stem and progenitor cells (HSPCs) expand under inflammatory stimulation. Lipopolysaccharide (LPS) induced acute inflammation (8) and increased IL-6 production by intestinal microbiota (25) promote expansion of Tet2^-/-^ HSPCs in mice while prolonged TNFα exposure *in vitro* increased the clonogenic capacity of Tet-2 deficient murine and TET2-mutant human bone marrow cells (26). More recently, IL-1α exposure was found to expand Tet2^+/-^ hematopoietic cells relative to wildtype cells while IL-1 receptor (IL-1R1) knockout mice failed to increase Tet2^+/-^ cells in response to IL-1α (27). Similarly, loss of IL-1R1 restored the hematologic abnormalities, myeloid-lymphoid balance and systemic inflammation observed in Tet2-deficient mice (28). These studies underscore the importance of inflammatory cytokine signaling in driving TET2 dependent CH. However, the contribution of smoking in promoting clonal expansion of TET2 mutant cells has not been explored.

Cigarette smoking exerts a severe oxidative and inflammatory insult upon the respiratory system leading to the upregulation of several pro-inflammatory cytokines. Smoking causes systemic inflammation and is an independent risk factor for CHIP (11, 12). The inflammatory milieu caused by smoking on the clonal outgrowth of specific CHIP mutations has not yet been fully elucidated.

In this study we tested the impact of a two-month nose-only exposure on the causal relationship between smoking and CH. We assessed hematopoietic stem cell numbers and gene expression profiling in wildtype mice. Then, in competitive repopulation assays we tested how smoke exposure affects the clonal expansion of two common mutations seen in hematologic malignancies and CHIP, JAK^V617F^ and TET2 loss-of-function. We hypothesized that CHIP mutant hematopoietic stem and progenitor cells are more resistant to inflammatory stimuli rendered by smoking than their normal counterparts which leads to their selective expansion in the context of smoke exposure.

## Materials and methods

### Mice

Wildtype C57BL/6J (CD45.2) (Stock No. 000664) mice at 8 weeks of age were purchased from the Jackson Labs for cigarette smoke exposure. The conditional knockout Jak2v617f^fl/+^ mice were a kind gift from Dr. Ann Mullaly and were crossed to the Vav-iCre mice from Jackson labs (Stock No. 008610) to generate Jak2^V617F^ mutant mice for bone marrow transplants. Tet2^-/-^ mice (Stock No. 023359) and C57BL/6.SJL (CD45.1) (Stock No. 002014) mice were purchased from the Jackson labs. Mice were housed in specific pathogen-free facilities at the University of California, Irvine and maintained on a 12-hour light/dark cycle. All animal procedures were performed under the approval of the Institutional Animal Care and Use Committee at the University of California, Irvine.

### Cigarette smoke exposure

Exposures were performed at the Air Pollution Health Effects Laboratory at the University of California, Irvine. Aerosols were generated using a 2-s puff with a 60-s interval between puffs (35 mL puff volume) based on the ISO standard cigarette puff protocol (ISO 3308:2012) using a custom-built smoking system. This system uses a peristaltic pump to draw in aerosolized smoke from a lit combustion cigarette (1R6F Certified Reference Cigarette, Center of Tobacco Reference Products (CTRP), University of Kentucky) and directs it into a nose-only exposure manifold (In-Tox Products, Moriarty, NM, USA). Control mice were exposed concurrently with the combustion cigarette exposure to air purified over potassium permanganate-impregnated alumina beads, activated carbon, and high-efficiency particulate air (HEPA) filters. The nose-cone inhalation exposure system is advantageous over whole-body exposure systems because it minimizes contamination of fur and potential non-relevant exposure due routine grooming that would occur in a whole-body exposure regime. During exposures, animals were held in individual exposure tubes that were connected to the exposure manifold and positioned with just the snout exposed to the exposure atmosphere. Exhaust ports surrounding each nose cone were under slight negative pressure which directs the flow of fresh aerosol to the animal’s breathing zone and exhausts exhaled air thereby minimizing the potential for re-breathing secondary vapor and preventing CO_2_ buildup. Exposures occurred 2 hours per day, 4 days/week, for 8 weeks. Between exposures, the mice were housed 4 per cage in an atmosphere-controlled room on a 12-hr light/dark cycle in an Association for the Assessment and Accreditation of Laboratory Animal Care (AAALAC) accredited animal housing facility at the University of California, Irvine vivarium.

### Cigarette smoke extract

Aqueous cigarette smoke extract (CSE) was prepared from mainstream cigarette smoke using an impinger and following a standard protocol (29). Briefly, mainstream smoke from one 2R4F research cigarette was drawn into serum-free media by continuous negative pressure and filter-sterilized to obtain 100% CSE. Volume/volume percentage (v/v %) dilutions were used for *in vitro* exposures.

### Bone marrow transplantation

Adult mice were euthanized before bone marrow harvesting. Euthanasia was performed using an overdose of isoflurane with the isoflurane concentration adjusted at 5% or greater and isoflurane exposure was continued until 1 minute after breathing stopped. Euthanasia was confirmed by cervical dislocation method. Recipient mice were lethally irradiated (single dose of 8Gy) and received 2×10^6^ whole bone marrow cells mixed in a 1:1 ratio from either air or smoke exposed mice and Pepcb/Boy CD45.1 mice *via* retro-orbital injection. Peripheral blood chimerism was assessed at regular intervals. For chimeric mice, lethally irradiated CD45.1/CD45.2 recipient mice were transplanted with 2x10^6^ unfractionated bone marrow mixed in a 1:1 ratio from Jak2^V617F^ and CD45.1 mice. Whole bone marrow from Tet2^-/-^ and CD45.1 wildtype mice were mixed in a ratio of 1:10 and a total of 3x10^6^ cells were injected into recipient mice. Chimeric mice were rested post-transplant before cigarette smoke exposure.

### Flow cytometry of mouse peripheral blood and bone marrow

Peripheral blood was collected from the saphenous vein and used to obtain complete blood counts using the automated cell counter machine (ABCVet Hemalyzer, scil). To determine peripheral blood chimerism, red blood cells were lysed using ammonium-chloride-potassium buffer stained with the anti-mouse antibodies against CD45.1 (clone A20), CD45.2 (clone 104), Ly6G (clone 1A8) and CD11b (M1/70). Bone marrow hematopoietic Pacific blue-conjugated anti-mouse antibodies against TER-119 (clone Ter119), CD3 (clone 17A2), Gr1 (clone RB6-8C5), CD11b (clone M1/70), B220 (RA3-6B2) were used to detect mature cells (Lineage cocktail). HSPC populations were detected by staining with CD34 (RAM34, BD Biosciences), CD16/32 (clone 93), c-Kit (clone 2B8), Sca-1 (clone D7), CD48 (clone HM48-1) and CD150 (clone TC15-12F12.2). For bromodeoxyuridine (BrdU) incorporation, mice were injected with BrdU at 1mg/kg and euthanized 16 hours later. Bone marrow cells were stained with cell surface markers, fixed, permeabilized and probed for BrdU following manufacturer’s instructions in the BrdU flow kit (BD Biosciences, San Jose, CA). For gamma-H2AX, stained, fixed and permeabilized cells were incubated with anti-H2A.X phospho (Ser139, clone 2F3) for 20 minutes at room temperature. All antibodies were purchased from BioLegend unless otherwise stated. Flow cytometry was performed on the Novocyte (ACEA Biosciences) at the UCI Immunology Core facility. Data were analyzed using FlowJo software (Tree Star Inc.).

### Colony formation assay

Colony formation assays were performed according to protocols provided by StemCell Technology and as described previously (20). Fresh peripheral blood mononuclear cells or whole bone marrow cells were incubated with increasing concentrations of cigarette smoke extract overnight in the presence of N-Acetylcysteine (100μM), as previously described (30), where indicated. Cells were washed thoroughly and plated in Methocult medium (H4230 andM3224, StemCell Technologies) containing human or mouse IL-3 at 10ng/ml, human or mouse SCF at 50ng/ml and human Epo at 20ng/ml. Colonies were counted 7-12 days later.

### Nanostring data analysis

Bone marrow cells from wildtype mice exposed to air or cigarette smoke were harvested and RNA was isolated using the RNeasy micro kit (Qiagen). WT/Jak2^V617F^ chimeric mice exposed to air or CS were euthanized within 24 hours of the last exposure and WT bone marrow cells were sorted on the BD FACSAria Fusion (BD Biosciences) by staining for anti-mouse CD45.1 and CD45.2 antibodies for RNA isolation. The nCounter Mouse PanCancer Immune Profiling Panel was used to analyze mRNA expression of immune function and inflammatory transcripts. Nanostring data was analyzed by ROSALIND^®^ (https://rosalind.bio/) (ROSALIND Inc, San Diego, CA). Data normalization was performed using the protocol from the nCounter advanced analysis software (NanoString Technologies). Abundance of various cell populations was calculated on ROSALIND using the Nanostring Cell Type Profiling Module. Differential gene expression was calculated following the nCounter^®^ Advanced Analysis protocol to identify targets with significantly increased or decreased expression. P-value adjustment was performed using the Benjamini-Hochberg method of estimating false discovery rates (FDR). Nanostring data are publicly available on ArrayExpress, accession number E-MTAB-13133.

### Statistical analysis

Statistical analyses were performed using GraphPad Prism (GraphPad Software, San Diego, CA). Data are presented as mean with error bars representing the SEM. Comparison between groups was performed using unpaired *t* test. For matched samples, paired t test was used to determine the *P* value. Two-way ANOVA was used to compare more than 2 groups and *P* value adjusted using Bonferroni’s correction.

## Results

### Cigarette smoke exposure induces secondary erythrocytosis in wildtype mice

**Figure 1:**
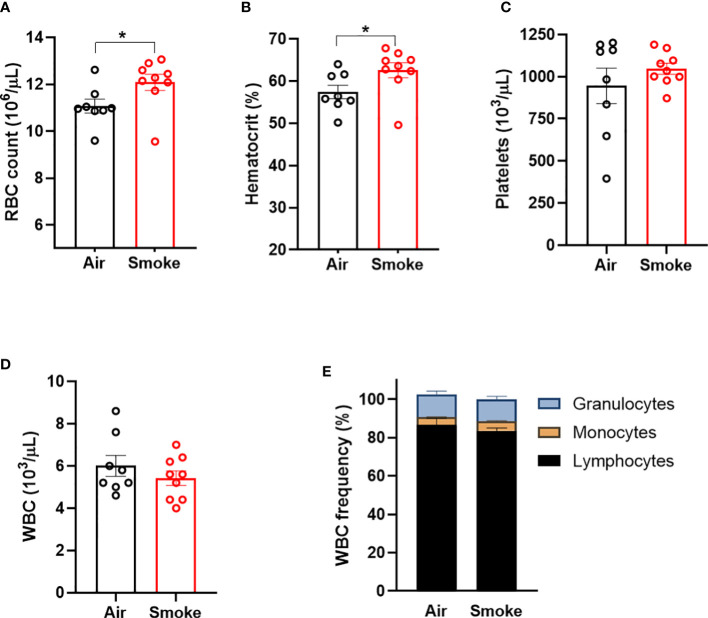
Combustible cigarette smoke exposure induces secondary erythrocytosis. Two months of smoke exposure in wildtype mice increased **(A)** red blood cell (RBC) count, and **(B)** hematocrit. **(C)** Platelet counts, **(D)** white blood counts (WBC), nor **(E)** white blood cell differentials were not affected by smoke exposure. Data are shown as mean ± SEM, n=8-9 mice/group. *p<0.05, unpaired student's t-test.

Wildtype C57BL/6J mice were exposed to nose-only inhalation of conventional combustible cigarette smoke (CS) for 2 hours per day, 4 days a week for 2 months. Cigarette smoke exposure in rodents can be carried out using either whole-body or nose-only exposure systems. The nose-only exposure allows for efficient and targeted exposure while limiting non-respiratory exposure routes such as ingestion and dermal absorption observed with whole-body exposure. Smoke exposure increased erythrocyte parameters such as red blood cell count (**Figure 1A**) and hematocrit (**Figure 1B**) in comparison to air exposed mice. However, platelet counts (**Figure 1C**), white blood cell counts and (**Figure 1D**) white blood cell differentials (**Figure 1E**) were not affected by smoke exposure. Body weight was increased by smoke exposure; however, heart, liver, and spleen weights were comparable between the two exposure groups (**Supplemental Figure 1**). These observations demonstrate that our smoke exposure model system replicates erythrocytosis observed in human smoking behavior.

### Cigarette smoke exposure induces inflammatory gene expression changes in BM cells

**Figure 2:**
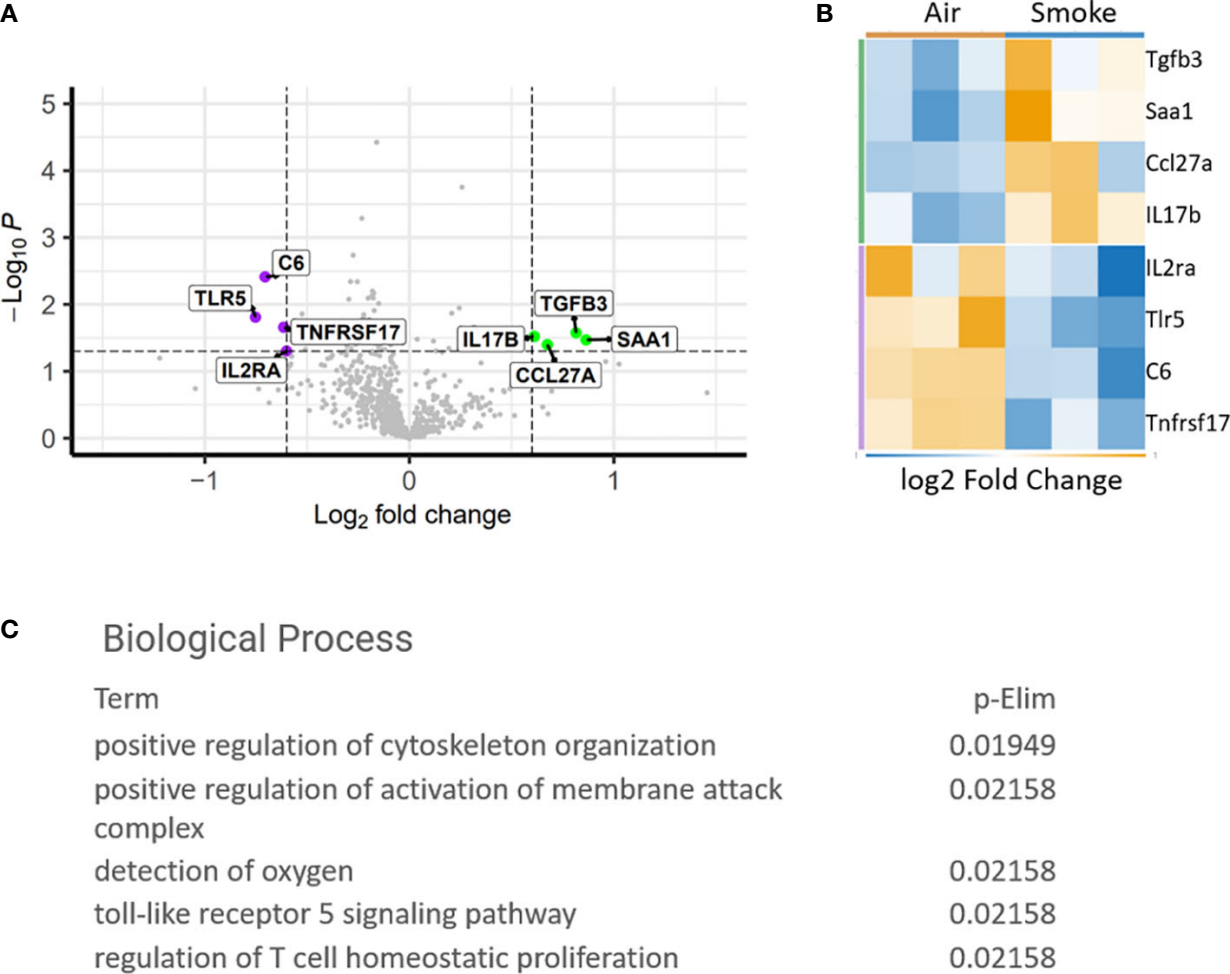
Differentially expressed genes in CS exposed bone marrow hematopoietic cells. **(A)** Volcano plot showing difference in expression between air and smoke exposure. Annotated genes are significantly differentially expressed, p<0.05. **(B)** Heatmap depicting the differentially expressed genes. **(C)** Gene ontology biological processes affected by smoke exposure.

To study the specific gene expression changes in hematopoietic cells exposed to CS, mice exposed to CS or air for 2 months were euthanized and bone marrow was harvested from the long bones for comparative expression analysis using the Nanostring PanCancer Immune Profiling Panel. Genes that were significantly upregulated include transforming growth factor beta 3 (Tgfb3), serum amyloid A1 (Saa1), C-C motif ligand 27 (Ccl27a) and interleukin 17 receptor A (IL17Ra). Interleukin-2 receptor alpha (IL2Ra), Toll-like receptor 5 (Tlr5), complement 6 (C6) and TNF receptor superfamily member 17 (Tnfrsf17) were found to be downregulated (**Figures 2A, B**). Biological processes associated with the dysregulated genes included T-cell homeostasis (**Figure 2C**). Saa1 and IL-17A are neutrophil mobilizing mediators, with IL-17A being a major inflammatory cytokine that drives pathological inflammation (31). Increase in IL-17Ra indicates upregulation of inflammatory responses and IL-17Ra has been shown to be involved in CS-induced chronic obstructive pulmonary disease (COPD) (32). IL2Ra or CD25 is expressed on T regulatory cells and its downregulation indicates an imbalance between inflammatory Th17 cells and immunosuppressive T regulatory cells and inability to suppress inflammation associated with CS (33). Interestingly, Saa1 can initiate polarization and maturation of IL-17A expressing cells and the combined upregulation of IL-17Ra and Saa1 serve as markers of persistent inflammation (34). Tgfb3 exerts a suppressive effect on B cells and also induces Th17 cells that display pathologic inflammation (35).

### Increased oxidative stress in hematopoietic stem cells exposed to cigarette smoke

**Figure 3:**
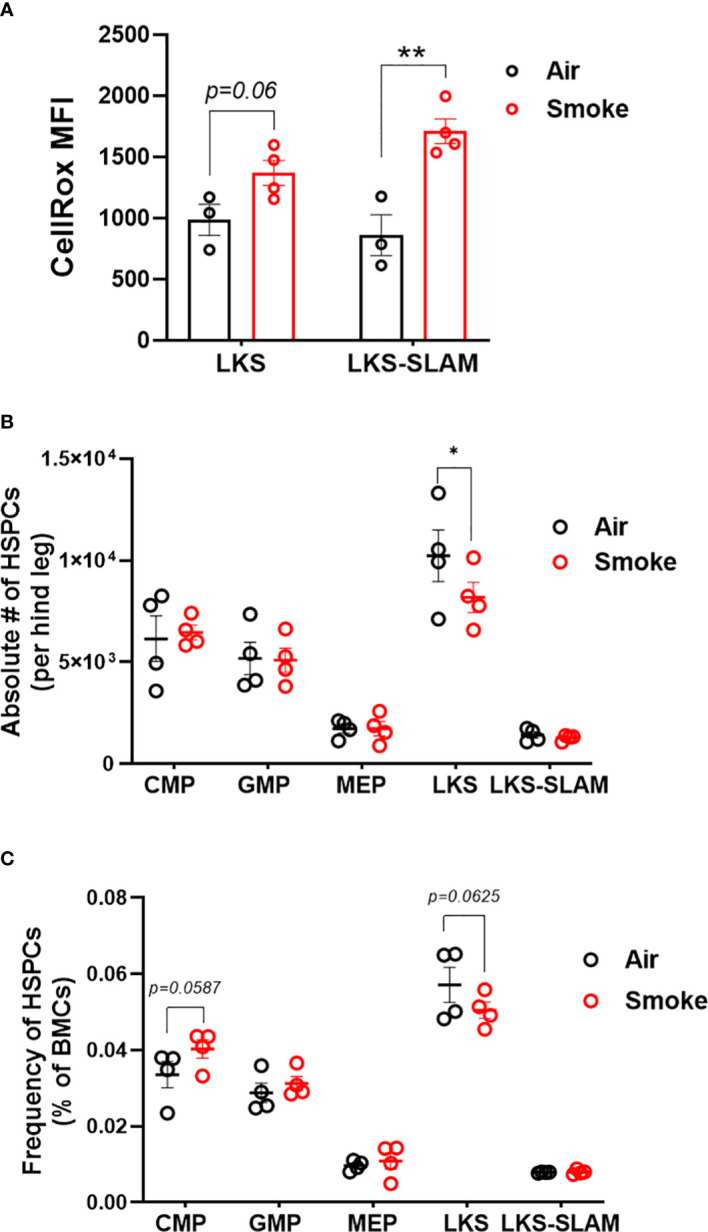
HSCs display oxidative stress upon smoke exposure. **(A)** Oxidative stress in LKS and LKS-SLAM cells as determined by CellRox staining. Absolute numbers **(B)** and frequencies **(C)** of hematopoietic stem and progenitor cell population after 2 months of exposure to nose-only smoke or air. Data are shown as mean ± SEM, n=3-4 mice per group. *p<0.05, **p<0.001, unpaired student’s t-test.

Next, we used flow cytometry to quantify the effect of CS on wildtype hematopoietic stem and progenitor cells (**Figure 3A**). Smoke exposure was associated with a significant increase in reactive oxygen species (ROS) within the lineage^-^, c-Kit^+^, Sca1^+^ (LKS) and LKS-CD150^+^, CD48^-^ (LKS-SLAM) or long-term HSCs (LT-HSCs) (**Figure 3B**) populations. Cigarette smoke lowered the absolute number of LKS cells in the bone marrow (**Figure 3C**) but did not significantly change the frequency of LKS cells (**Figure 3C**). We did not detect a statistical difference in the frequency nor absolute numbers of LKS-SLAM, or myeloid progenitor compartments (common myeloid progenitor CMP, granulocyte monocyte progenitor GMP, megakaryocyte erythroid progenitor MEP) when comparing air versus smoke exposed mice (**Figures 3B, C**).

### Smoke exposure does not compromise competitive repopulation ability

**Figure 4:**
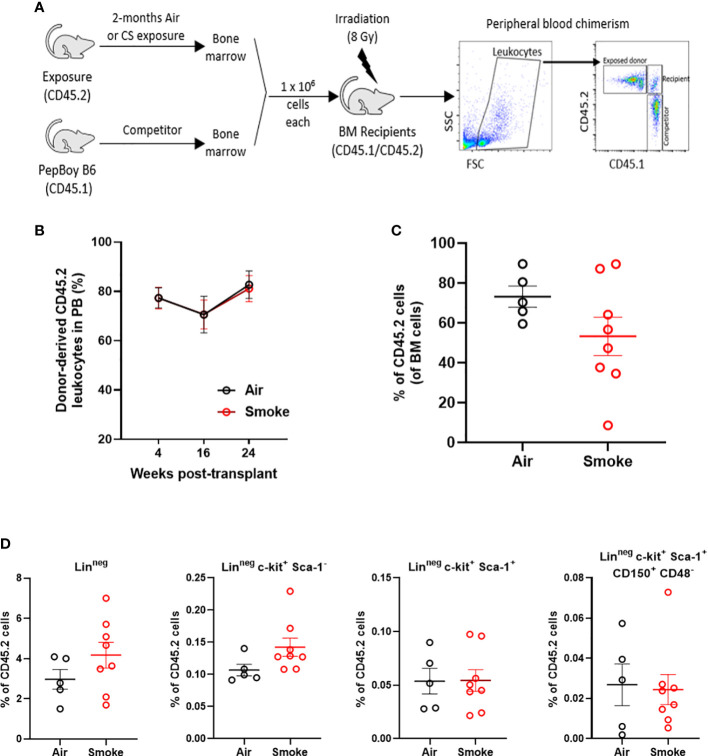
Smoke exposure does not compromise competitive repopulation ability. **(A)** Experimental design of competitive repopulation assay used. **(B)** contribution of CD45.2 donor cells to peripheral blood leukocytes of transplanted mice over time and **(C)** contribution of CD45.2 donor cells to bone marrow leukocytes at time of euthanasia, **(D)** frequencies of progenitor and stem cell populations gated on CD45.2 bone marrow cells. Data are shown as mean ± SEM, n=5-7 mice per group.

To functionally assess the impact of smoke exposure on hematopoietic stem cells we transplanted air-or smoke-exposed whole bone marrow cells along with equal numbers of unexposed cells into lethally irradiated mice and followed peripheral blood chimerism (**Figure 4A**). Competitive ability of bone marrow from mice exposed to smoke was equivalent to that of air exposed mice in primary recipients (**Figure 4B**). Six months after transplant, recipient mice were euthanized and their bone marrow was analyzed. The contribution of air and smoke exposed CD45.2 donor cells to total donor derived bone marrow leukocytes was equivalent (**Figure 4C**). To determine if bone marrow from smoke exposed mice display expansion or shrinkage of stem and progenitor cells, we quantified the frequency of lineage negative, c-kit^+^, Sca-1^-^ (contains myeloid progenitors), LKS, and LKS-SLAM of total CD45.2 cells (**Figure 4D**). We found no significant differences in the frequencies of these populations when comparing air versus smoke exposed donors.

### *In vitro* cigarette smoke extract reduces myeloid colony formation partially through oxidative stress

**Figure 5:**
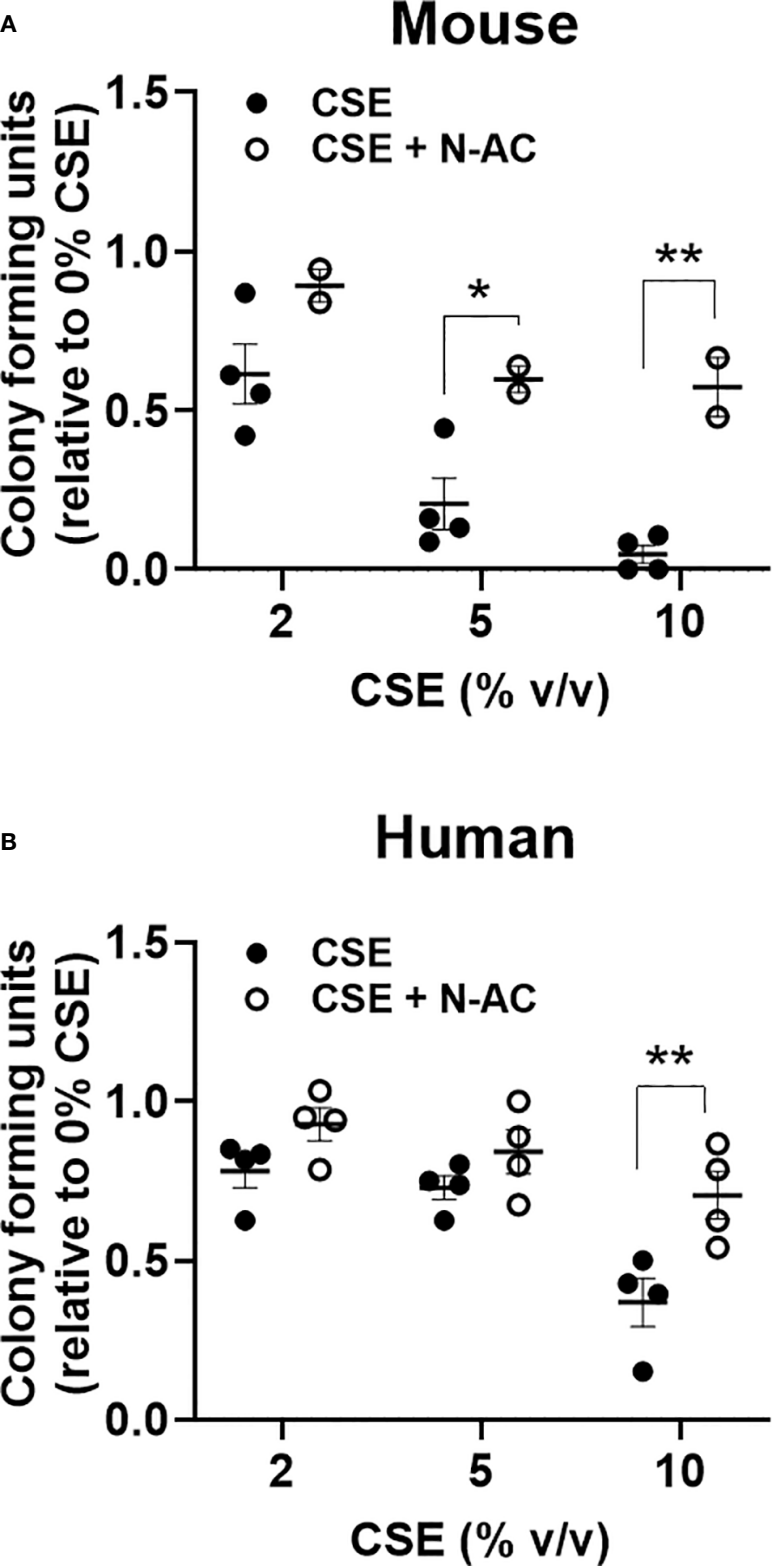
Acute *in vitro* exposure reduces myeloid colony formation and is rescued by the anti-oxidant N-Acetylcysteine. **(A)** Wildtype mouse bone marrow cells and **(B)** normal human peripheral blood mononuclear cells were incubated overnight with increasing concentrations of cigarette smoke extract +/- 100nM N-AC, washed twice, then plated in methylcellulose supplemented with SCF, IL-3, and EPO. Colonies were enumerated 7-10 days later. *p<0.05, **p<0.01 unpaired student’s t-test.

To evaluate the impact of an acute *in vitro* smoke exposure on hematopoiesis bone marrow from wildtype mice and peripheral blood from normal human controls were cultured overnight with 0, 2, 5, or 10% cigarette smoke extract (CSE), washed prior to plating on methylcellulose, and colonies were counted 7-12 days later. We observed a decrease of both mouse (**Figure 5A**) and human (**Figure 5B**) myeloid colony formation with overnight exposure to CSE. To determine if this effect was mediated by oxidative stress we included the anti-oxidant N-Acetylcysteine (N-AC) in the overnight CSE culture. N-AC rescued the suppressive impact of CSE on colony formation, suggesting that CSE’s suppressive effect is mediated *via* oxidative stress (**Figures 5A, B**).

### Smoke exposure enhances competitive ability Jak2^V617F^ mutant cells

**Figure 6:**
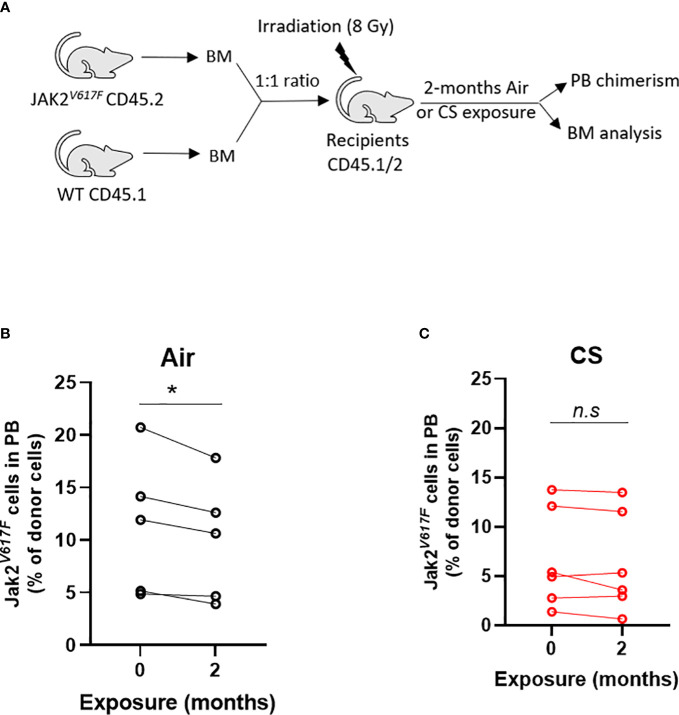
Smoke exposure maintains survival of JAK2^V617F^ mutant cells. **(A)** Experimental design. Change in circulating Jak2^V617F^ cells of **(B)** air and **(C)** cigarette smoke exposures. Data are shown as percentage chimerism before and after exposure. n=4-6 mice per group. *p<0.05, paired t-test.

Next, we tested whether cigarette smoke impacts the competitive ability of Jak2^V617F^ mutant cells in competitive repopulation assays. C57BL/6J mice were lethally irradiated and transplanted with equal numbers of whole bone marrow from Jak2^V617F^ and WT donors (1:1) (**Figure 6A**). This knock-in model of Jak2^V617F^ does not display a competitive advantage in a lethally irradiated bone marrow transplant setting and we expect to observe decreasing percentage of Jak2^V617F^ cells in the peripheral blood over time. Mice were rested post-transplant and then exposed to 2 months of combustible cigarette smoke *via* nose-only inhalation. The percentage of mutant cells was low (2-20%) to mimic clonal hematopoiesis without an overt myeloproliferative neoplasm phenotype. Smoke exposure in transplanted mice slightly increased red blood cells, p=0.055, and showed a trend towards increased hematocrit, and leukocytes (p<0.1) (**Supplemental Figures 2A-D**). There was no significant increase in spleen to body weight ratio (**Supplemental Figure 2E**) nor liver weight (**Supplemental Figure 2F**). As expected, mice exposed to air demonstrated a decline in Jak2^V617F^ mutant cells in the peripheral blood over time, however in contrast smoke exposed mice maintained circulating Jak2^V617F^ levels before and after exposure (**Figure 6B**).

### Smoke exposure increases HSC DNA damage and proliferation

**Figure 7:**
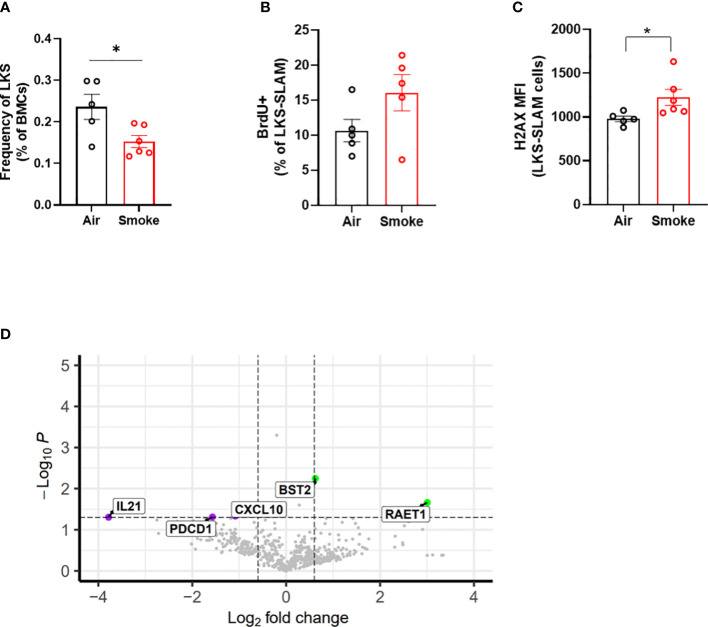
Cigarette smoke increases DNA damage in HSCs. **(A)** LKS frequency, **(B)** BrdU incorporation and **(C)** ΥH2AX MFI in LKS-SLAM cells from WT : Jak2^V617F^ transplanted mice. **(D)** Volcano plot of relative gene expression in WT cells sorted from air and smoke exposed transplanted mice. Data are shown as mean ± SEM. *p<0.05, unpaired student’s t-test.

We compared the impact of CS on wildtype and Jak2^V617F^ mutant cells in transplanted and exposed mice. We observed a significant reduction in the frequency of total LKS cells (containing Jak2^V617F^ and wildtype cells combined) in transplanted mice exposed to smoke (**Figure 7A**). To identify the relationship between smoke exposure and HSC cycling, air- and smoke-exposed mice were injected with bromodeoxyuridine (BrdU,1mg/kg) sixteen hours prior to euthanasia to capture proliferation. While it was not technically feasible to assess the phenotype of mutant and wildtype cells separately with this experimental protocol, we were able to observe specific smoke induced changes in all bone marrow cells, irrespective of genotype. We observed a trend towards increased cell division in LKS-SLAM cells exposed to smoke as seen by the increased percentage of BrdU positive cells (**Figure 7B**), supporting the notion that CS induces low grade chronic inflammation. We also documented increased gamma-H2AX fluorescence in LKS-SLAM cells exposed to smoke indicating DNA damage (**Figure 7C**).

### Smoke exposure induces inflammatory gene expression in wildtype bystander cells in mice containing Jak2^V617F^ cells

To explore how smoke exposure impacts gene expression of wildtype cells when co-existing with a small population of Jak2^V617F^ cells, we compared inflammatory gene expression in wildtype whole bone marrow cells sorted from air and smoke exposed Jak2^V617F^/WT chimeric mice. The differentially expressed genes in transplanted mice showed overlap with biological processes related to T cell functions as observed in the untransplanted mice. IL-21 is involved in the generation of Th17 cells (36) while programmed cell death 1 (Pdcd1) plays a role in Treg differentiation (37). Cxcl10 is an inflammatory cytokine implicated in COPD in smokers (38). Interestingly, we also identified overexpression of retinoic acid early transcript 1 (Raet1) and bone marrow stromal antigen 2 (Bst2) specific to the transplanted mice (**Figure 7D**). Raet1 is a ligand for the cytotoxic lymphocyte activating receptor (NGKD) and plays a major role in NK cell function, and is expressed only under cellular stress conditions such as oxidative stress, DNA damage and hyperproliferation (39). Bone marrow stromal antigen 2 (Bst2) is involved in the growth and differentiation of B cells but can also be induced on other cells by the IFN pathway. Interferon gamma (IFNg) stimulation of HSCs induces surface expression of Bst2 leading to displacement of HSCs from their quiescent niche followed by cell cycle activation (40).

### Jak2^V617F^ renders cells resistant to the suppressive effects of CSE in colony formation assays

**Figure 8:**
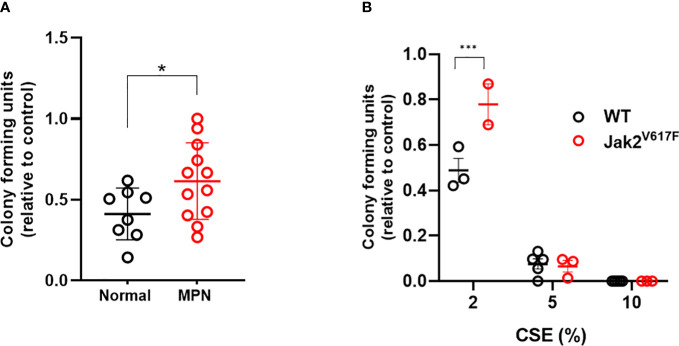
MPN cells are resistant to the suppressive effects of CSE. **(A)** Human peripheral blood mononuclear cells were incubated overnight with 0 or 2% CSE, then washed prior to plating in methylcellulose supplemented with SCF, IL-3, and EPO, and enumerated 7-10 days later. **(B)** Mouse bone marrow cells were incubated overnight in 0, 2, 5 or 10% CSE, washed and then plated in methylcellulose. Data are shown as mean ± SEM. *p<0.05, unpaired student’s t-test. ***p<0.001, 2-way ANOVA with Bonferroni’s correction.

To determine whether JAK2^V617F^ protects hematopoietic progenitors from the suppressive ex vivo effects of acute smoke exposure, we performed methylcellulose colony forming assays on peripheral blood mononuclear cells from MPN patients and normal controls incubated in cigarette smoke extract (CSE). There was significant difference in the clonogenic capacity of CSE exposed cells obtained from MPN patients compared to normal subjects (**Figure 8A**) suggesting that JAK2^V617F^ renders cells resistant to acute ex vivo smoke exposure. Parallel experiments using Jak2^V617F^ and wildtype mouse bone marrow cells yielded similar results (**Figure 8B**). Together, with *in vivo* exposure to smoke preserving the competitive ability of Jak2^V617F^ and *in vitro* resistance suggest that smoke exposure promotes Jak2^V617F^ selection.

### Cigarette smoke promotes selective outgrowth of Tet2^-/-^ cells

**Figure 9:**
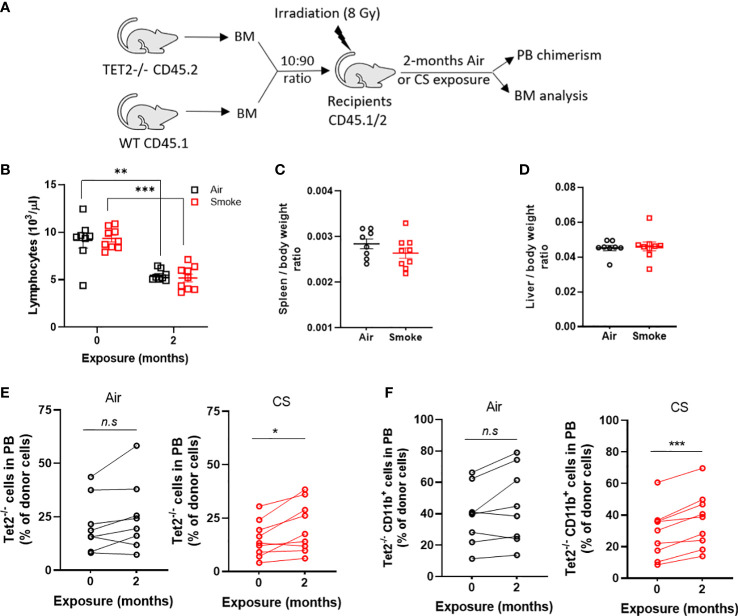
Cigarette smoke promotes the selective expansion of Tet2^-/-^ cells. **(A)** Experimental design. **(B)** Deceased lymphocytes in air and smoke exposed mice over time. **(C)** Spleen and **(D)** liver body weight ratio in exposed mice. Change in peripheral blood chimerism of Tet2^-/-^ **(E)** leukocytes and **(F)** myeloid specific cells. **(B–D)** Data are shown as mean ± SEM. n=8-9 mice per group. **p<0.01, ***p<0.001, unpaired student’s t-test. (E, F) Data are shown as percentage chimerism before and after exposure, *p<0.05, ***p<0.001, paired t-test.

Next, to investigate whether smoke selects for hematopoietic cells with other common mutations seen in CHIP and hematologic malignancies, we exposed mice transplanted with a mixture of Tet2^-/-^ and WT cells to air and CS for two months. Since Tet2^-/-^ cells display a significant selective advantage in competitive repopulation assays we utilized a low input ratio of Tet2^-/-^ cells (1:10 ratio) to enable assessment of changes in mutant cell populations following extrinsic stressors (**Figure 9A**). Mice were rested for several months post-transplant and then exposed to two months of CS or air *via* nose-only inhalation. Mice in both air and smoke groups displayed a reduction in lymphocytes with time due the myeloid-lymphoid imbalance that is characteristic of Tet2-deficiency (**Figure 9B**). Spleen and liver weight were not affected by smoke exposure in Tet2^-/-^ transplanted mice (**Figures 9C, D**). The contribution of Tet2^-/-^ donor cells to total leukocytes (**Figure 9E**) as well as myeloid cells (**Figure 9F**) increased during the two-month exposure in the CS group but remained stable in the air group.

## Discussion

In this study, we performed physiological cigarette smoke inhalation exposures in mice to demonstrate that CS can promote the selective expansion of specific CHIP mutant clones. Our results provide evidence that environmental exposure to cigarette smoke can promote the expansion of pre-existing Jak2^V617F^ and Tet2-deficient clones. The mechanisms by which CS selects for clonal selection is likely *via* induction of low-grade chronic inflammation and oxidative stress. Tet2^-/-^ cells have been shown to expand under several different environmental stressors including inflammatory stimulation (8), space flight (41), atherogenic diet (5), hyperglycemia (42), and sleep fragmentation (10). Many of these external stressors do not occur independently but can co-exist due to lifestyle behaviors. It would be important to assess the synergistic or additive effect of co-stressors along with CS on the augmentation of CH. The short course of the exposure (2 months) allowed us to assess the immediate impact of active smoke exposure, however prolonged effects of a remote smoking exposure was not assessed. Specifically, we did not address whether smoke exposure may increase the likelihood of progression from Tet2^-/-^ CHIP to a bona fide hematologic malignancy. Likely the impact of a remote exposure has a long-lasting effect on clonal selection supported by the increased incidence of CHIP in people with previous history of smoking.

We observed a diminished pool of phenotypically defined HSPCs (LKS cells) in both our untransplanted as well as transplanted mouse models. It is likely that this reduction in the LKS population is mediated *via* direct effects of nicotine through the α7 nicotinic acetylcholine receptor (α7nAChR). Acetylcholine has been recently described to control steady-state hematopoiesis and preserve stem cell quiescence (43, 44) while, CHRFAM7A, a dominant negative inhibitor of α7nAChR, leads to an increased reservoir of HSCs in the bone marrow (45). Other smoke exposure studies in mice have also described reduced HSPCs as early as 3 days post exposure (46) and as long as 9-month exposures (47). We have also observed LKS suppression in mice exposed to electronic (E-) cigarette vape containing nicotine (48). HSCs from smoke exposed animals showed increased reactive oxygen species (ROS) and evidence of DNA damage, and cycling compared to air exposed mice. Inflammatory and oxidative stressors are known to drive cycling and myeloid bias of HSCs implying that the different constituents of cigarette smoke can induce opposing effects on HSCs. Our findings of a decreased LKS compartment, increased ROS, DNA damage, and cycling of the HSC compartment in smoke exposed animals did not translate into an observable defect in competitive repopulation ability in primary recipients. It is possible that a short *in vivo* exposure to smoke leads to subtle functional defects in HSC that would only be revealed with expanded proliferative stress such as secondary or tertiary transplants.

We identified a smoke induced pro-inflammatory gene expression signature in bone marrow hematopoietic cells from wildtype mice. We found evidence for increased inflammation involving both the myeloid and lymphoid lineages. Serum amyloid A (Saa1) was increased which is an acute-phase protein that plays a role in the initiation and maintenance of inflammation. Saa1 leads to neutrophilia and production of inflammatory cytokines TNF-α, IL-6 and IL-17 (34). TNF-a and IL-1b also induce the production of Ccl27a, a cytokine involved in T-cell inflammation (49), which we also observed to be upregulated by smoke exposure. Tgfb3, another upregulated gene, plays a pro-inflammatory role by inducing pathogenic Th17 cells (35). We found downregulation of genes that are involved in dampening of an immune response, including IL-2ra. IL-2ra or CD25 is highly expressed on regulatory T-cells (Tregs) and its downregulation indicates immune dysfunction. Tnfrsf17 belongs to the Tnf superfamily receptors and is required for B cell development and immune responses. Tnfrsf17 was previously identified as a blood-based smoke exposure gene signature in humans (50), corroborating our observations in mice.

We also quantified changes in gene expression in wildtype cells co-existing in an environment containing Jak2^V617F^ mutant cells. Our intent was to investigate how the presence of Jak2^V617F^ may modulate or augment the impact of smoke exposure on inducing a pro-inflammatory gene signature in bystander wildtype cells. While the B cell abundance score was suppressed in both untransplanted and transplanted mice following smoke exposure, the presence of Jak2^V617F^ cells increased the cell abundance of neutrophils, indicating an inflammatory phenotype. However, we cannot rule out the possibility that these mice were lethally irradiated and thus display distinct changes compared to naïve wildtype mice. We also observed different gene expression signatures in the wildtype mice and Jak2^V617F^/WT transplanted mice exposed to cigarette smoke. One reason for this discrepancy could be the age of the mice at the time of cell collection. Wildtype mice were 4 months old at the end of the exposure while the Jak2^V617F^/WT transplanted mice were between 12 to 15 months old when exposures were completed and bone marrow was harvested. It is possible that the aging bone marrow environment in the older mice influences inflammatory gene expression differently upon smoke exposure. Another reason for the observed differences could be the impact of whole-body irradiation and the consequent inflammation and stress hematopoiesis associated with bone marrow transplant. Inflammatory processes and regulatory immune mechanisms that are impacted by radiation could alter the gene expression signature induced by cigarette smoke in transplanted mice. Finally, we also speculate that the presence of Jak2^V617F^ mutant cells in the bone marrow environment could result in altered inflammatory responses of the bystander wildtype cells. Studies have shown that the presence of JAK2^V617F^ and MPL^W515L^ mutant cells leads to aberrant cytokine production in bystander non-malignant cells (51).

Jak2^V617F^ mutant progenitors utilize DUSP1 activity to resist inflammation induced DNA-damage and tightly regulate ROS to promote their survival in an inflammatory environment (52). We previously demonstrated that *JAK2^V617F^* cells are resistant to TNF-α (20). We speculate that the survival of Jak2^V617F^ cells from transplanted mice and from MPN patients in a cigarette smoke environment display increased survival due to their ability to evade apoptotic cues.

Clonal expansion of Tet2^-/-^ hematopoietic cells has been investigated under different inflammatory stressors (8, 10, 28) but the impact of cigarette smoke on Tet2^-/-^ selection has not been studied. We identified a significant increase in Tet2^-/-^ cells upon CS exposure demonstrating that CS exerts a selection pressure for the outgrowth of Tet2^-/-^ cells. However, when determining systemic inflammation using plasma samples from CS and air-exposed mice, we did not observe differences in TNF-α or IL-6 (data not shown). It is possible that changes in immune populations and inflammatory cytokines in the local bone marrow environment exert a selection pressure. Future studies are aimed at investigating the inflammatory phenotype of individual bone marrow immune cell types. An important caveat to note is that the *Tet2-/-* mouse model is lacking TET2, whereas CHIP associated TET2 mutations in humans are usually heterozygous point mutations.

Mutant Asxl1 has been specifically associated with smoking suggesting that CS-induced inflammation promotes the outgrowth of Asxl1 mutant clones (12). Since, the type of stressor is proposed to determine the type of clone that will expand, we hypothesize that Asxl1 mutants will be strongly responsive towards CS exposure and we are currently investigating this using bone marrow transplanted mice containing mouse Asxl1 knockout cells.

In summary, we demonstrate that short-term exposure to cigarette smoke is sufficient to stimulate the expansion of Tet2-loss-of-function hematopoietic cells and maintain survival of Jak2^V617F^ clones. The underlying mechanisms driving clonal expansion need to be investigated with possible mechanisms including HSC resistance to apoptosis, increased HSC differentiation and inflammatory phenotype of mature mutant cells.

## Data availability statement

The data presented in the study are deposited in the ArrayExpress repository, accession number EMTAB-13133.

## Ethics statement

The studies involving human participants were reviewed and approved by Institutional Review Board of the University of California, Irvine. The patients/participants provided their written informed consent to participate in this study. The animal study was reviewed and approved by Institutional Animal Care and Use Committee at the University of California, Irvine.

## Author contributions

GR developed experimental plan, performed experiments, analyzed data, created figures, wrote the manuscript; JC and NM performed experiments, analyzed data, created figures and edited the manuscript; TT, AH, JM, BB, DH performed experiments; EM analyzed data; MK and AF developed experimental plan, oversaw research, analyzed data, and edited the manuscript.
