# Supplementary material for: Cigarette smoke stimulates clonal expansion of Jak2V617F and Tet2-/- cells
Source: Front Oncol. 2023 Jul 21;13:1210528. doi: 10.3389/fonc.2023.1210528 (PMC10401270; doi:10.3389/fonc.2023.1210528)
Supplement: Supplementary file 1 [file DataSheet_1.docx]

Supplementary Material

**Cigarette Smoke Stimulates Clonal Expansion of Jak2^V617F^ and Tet2^-/-^ Cells**

Gajalakshmi Ramanathan^1*^, Jane H. Chen^1*^, Nitya Mehrotra^1*^, Tiffany Trieu^1^, Aaron Huang^1^, Eduard Mas ^1^, Jessica E. Monterrosa Mena^2^, Bishop Bliss^2^, David A. Herman^2^, Michael T. Kleinman^2^ and Angela G. Fleischman^1,3^

^1^Department of Medicine, Division of Hematology/Oncology, University of California, Irvine, Irvine, CA, United States

^2^Department of Environmental and Occupational Health, Program in Public Health, Susan and Henry Samueli College of Health Sciences, University of California, Irvine, Irvine, CA, United States

^3^Chao Family Comprehensive Cancer Center, University of California, Irvine, Irvine, CA, United States

*Equal contribution

**Correspondence:**Angela Fleischman
Email: agf@uci.edu

## Supplementary Figures

**Supplementary Figure 1**. Impact of two months of smoke exposure on body and organ weights of wildtype mice. (A) Body weight, (B) heart weight, (C) spleen weight, (D) spleen/body weight ratio, and (E) liver weight. Data are shown as mean±SD, n=8-9 mice/group. *p<0.05, unpaired student’s t-test.

**A.**

**B.**

**C.**

**D.**

**E.**

**B.**

**C.**

**D.**

**Supplementary Figure 2.** Peripheral blood counts and spleen and liver weights in mice Jak2^V617F^:WT chimeric mice. (A) Red blood cells, (B) hematocrit, (C) leukocytes, and (D) platelets in Jak2^V617F^:WT transplanted air and smoke-exposed mice. (F) Spleen and (G) liver to body weight ratio in air and CS exposed mice. Dat are shown as mean ± SEM, n=4-6 mice per group.

**A.**
